# ScFv-h3D6 Prevents Bapineuzumab-Induced Hemorrhagic Events in the APP23 Mouse Model of Alzheimer’s Disease

**DOI:** 10.3390/biom15111602

**Published:** 2025-11-15

**Authors:** Silvia Lope-Piedrafita, Gabriel Serra-Mir, Paula Melón, Anna Bonaterra, Mar Hernández-Guillamon, Sandra Villegas

**Affiliations:** 1 Unitat de Biofísica, Departament de Bioquímica i Biologia Molecular, Facultat de Medicina, Universitat Autònoma de Barcelona, 08193 Cerdanyola del Vallès, Spain; 2 Servei de Ressonància Magnètica Nuclear, Universitat Autònoma de Barcelona, 08193 Cerdanyola del Vallès, Spain; 3 Centro de Investigación Biomédica en Red-Bioingeniería, Biomateriales y Nanomedicina (CIBER-BBN), Universitat Autònoma de Barcelona, 08193 Cerdanyola del Vallès, Spain; 4 Protein Design and Immunotherapy Group, Departament de Bioquímica i Biologia Molecular, Facultat de Biociències, Universitat Autònoma de Barcelona, 08193 Cerdanyola del Vallès, Spain; 5 Neurovascular Research Laboratory, Vall d’Hebron Research Institute, Universitat Autònoma de Barcelona, 08035 Barcelona, Spain

**Keywords:** Alzheimer’s disease, amyloid-related imaging abnormalities, cerebral amyloid angiopathy, immunotherapy, magnetic resonance imaging, single-chain variable fragment

## Abstract

The occurrence of amyloid-related imaging abnormalities (ARIAs), found in clinical trials for Aβ-immunotherapy, has been related to the antibody’s effector function on glial activation by the Fc portion of the antibody. The use of single-chain variable fragments (scFv) has been proposed as a safer therapeutic strategy. Here, the effects of the mice format of bapineuzumap (mAb-m3D6) and its scFv (scFv-h3D6) on the occurrence of ARIAs in the APP23 mouse model of Alzheimer’s disease (AD) and cerebral amyloid angiopathy (CAA) have been addressed by magnetic resonance imaging (MRI). Results are supported by histological and/or biochemical determinations. Aged APP23 mice showed a significantly higher number of microhemorrhages than non-transgenic mice. mAb-m3D6 produced an increase in the number of new hemorrhagic events, mainly in the cortex, whereas scFv-h3D6 did not. Both mAb-m3D6 and scFv-h3D6 reduced Aβ levels by the same extent. Axonal/myelin damage was found in the frontal corpus callosum of APP23 mice, which did not recover after treatment. In conclusion, the scFv-h3D6 format appears safer than the full-length mAb in the APP23 model of AD and CAA. This finding is highly relevant in light of the new FDA- and EMA-approved mAbs, which exclude *APOEε4* allele carriers due to the occurrence of hemorrhages.

## 1. Introduction

Alzheimer’s disease (AD) is a neurodegenerative condition that affected most of the over-50 M people having dementia worldwide in 2020 [1]. At the histological level, the AD brain is characterized by the occurrence of extracellular β-amyloid (Aβ) plaques and intracellular neurofibrillary tangles (NFTs), composed of hyperphosphorylated tau protein [2]. According to the amyloid hypothesis, the early accumulation of Aβ peptides in the brain triggers the pathogenic cascade, leading to tau hyperphosphorylation, microtubule instability, neuroinflammation, synaptic loss, and neuronal death, though other pathophysiological factors are also likely to be involved [3,4,5].

The beneficial effect of Aβ-immunotherapy was first demonstrated in the AN1792 (Janssen & Wyeth, MO, USA) phase 2a clinical trial, consisting of the testing of active vaccination with the full-length, fibrillar Aβ_1-42_ peptide and QS21 adjuvant. Despite the fact that the treatment was discontinued in 2002 after a subset of immunized patients suffered subacute meningoencephalitis [6], the respondents in the study maintained low but detectable, sustained anti-AN1792 antibody titers and demonstrated reduced functional decline 4.6 years after immunization [7]. The autopsies of some respondents showed the presence of T-lymphocyte meningoencephalitis and infiltration by macrophages in the cerebral white matter, apart from the beneficial clearance of Aβ plaques and the lack of plaque-associated dystrophic neurites and astrocyte clusters [8]. Therefore, Fc-mediated phagocytosis of Aβ by microglia was proposed as the clearance mechanism, as well as the trigger of inflammatory processes.

The current generation of Aβ active vaccines is in phase 2 clinical trials and consists of N-terminal Aβ sequences (i.e., 1-5) because the N-termini is solvent-exposed in the 3D structures of both trimers (1-40, totally solvent-exposed) and dimers (1-42, partially exposed) of the Aβ peptides building amyloid fibrils [9,10]. This rationale is also being used in the development of some of the monoclonal antibodies (mAbs) for passive Aβ-immunotherapy [4]. The main advantage of passive versus active immunotherapy is that treatment can be discontinued in the event that a severe side-effect occurs. Bapineuzumab (Janssen/Pfizer), the humanized form of mAb-m3D6, was the first anti-Aβ (1-5) mAb to reach phase 3 clinical trials. In a phase 1 study enrolling patients with mild-to-moderate AD, three out of ten patients in the highest-dose group developed transient signal abnormalities on T2-weighted/fluid attenuation inversion recovery (FLAIR) sequences, a variant of magnetic resonance imaging (MRI), some weeks after a single dose of bapineuzumab [11]. In subsequent phase 2 studies, such signal abnormalities were detected in 9.7% of the bapineuzumab-treated patients, with a higher frequency in APOEε4 carriers and higher dose groups [12]. These studies came up with the term amyloid-related imaging abnormalities, ARIAs, to explain these signal abnormalities, especially as vasogenic edema or ARIA-E. As opposed to cytotoxic edema, vasogenic edema stems from an increase in extracellular fluid volume due to the increased permeability of brain capillary endothelial cells to serum proteins. Also in phase 1, other MRI alterations, specifically on long-echo-time gradient-refocused echo (T2*-GRE) sequences, attributable to hemosiderin deposits, were interpreted as hemorrhagic events and termed ARIA-H. Indeed, both abnormalities arise after the disruption of vessel integrity, leading to a leakage of proteinaceous fluid (ARIA-E) and red blood cells (ARIA-H) [13]. Results from two phase 3 studies showed the occurrence of edema in 15.3% of the APOEε4 carriers who received the lowest dose of bapineuzumab, concretely in 11.4% of APOEε4 heterozygotes and 27.3% of APOEε4 homozygotes [14]. In the non-carrier study, the occurrence of ARIA was also dose-dependent, ranging from 4.2% in the lowest-dose group to 14.2% in the highest-dose group. These adverse effects, together with the lack of effect on cognitive decline, led to the discontinuation of bapineuzumab development in 2012 [14]. The lack of success with bapineuzumab was attributed to the stage of the enrolled patients, mild-to-moderate, being too late. Hence, most recent studies are performed at the prodromal or early stages [15].

An interesting observation is that antibodies against the N-terminus are likely to mobilize less Aβ from neuritic plaques than antibodies against the mid-region or the C-terminal region of the peptide do, concurring with the rationale behind the aforementioned 3D structures for Aβ oligomers and fibrils [9,10]. This could be one of the reasons why lower rates of ARIA-E have been reported in trials with antibodies against the mid-region or the C-terminal region of the peptide [16].

Upon opsonization of Aβ-oligomers and fibrils, the Fc portion of the therapeutic antibody is thought to be responsible for the activation of microglia, the subsequent inflammatory response, and a cerebral amyloid angiopathy (CAA) manifested as cerebral vascular edema and hemorrhages [17,18]. CAA and AD pathology frequently co-occur in the same brain, presumably because Aβ is pathogenic in both; however, the mechanisms by which CAA and AD lead to brain injury are different [16].

The Fc portion of the antibody is engaged in affinities and specificities for Fc receptors, such as Fc-αRs and Fc-γRs, and complement the protein C1q. Fc-γRs are expressed on several cell types, including microglia. In hopes of reducing the antibody’s effector function on microglial activation, Pfizer/Janssen developed an Fc-engineered variant of bapineuzumab, AAB-003, but no improvement was achieved [19]. The use of single-chain variable fragments (scFv) and F(ab’)2 fragments, fragments lacking the Fc portion and therefore unable to directly activate microglia, have been proposed to prevent ARIA and so to constitute a safer therapeutic strategy. Interestingly, non-Fc-mediated clearance of antibody fragments has long been known [18]. Specifically, F(ab’)2 fragments of 3D6 led to the clearance of 45% of the amyloid deposits, the same extent as the full-length 3D6-mAb. Therefore, the direct disruption of plaques is one of the mechanisms involved in the antibody-mediated clearance of amyloid-beta deposits in vivo, in addition to Fc-dependent phagocytosis. In this sense, one of the main players in AD physiopathology is apoE, with reported different capabilities of amyloid clearance depending on the isoform [20]. This concurs with the incidence of ARIA in *APOEε4* allele carriers in the clinical studies mentioned above. For an update on the antibody fragments for neurological diseases, please see [21].

The scFv-h3D6 is a bapineuzumab (AAB-001) derivative that has been proven efficient and safe in a triple-transgenic mouse model of AD (3xTg-AD) [22,23,24,25,26,27,28]. It crosses the blood–brain barrier and it is internalized by the Aβ peptide-containing neurons before engulfment of the scFv-h3D6/Aβ complex by glial cells [29]. At the early stages of the disease, a single intraperitoneal administration of scFv-h3D6 prevented neuronal loss in the hippocampus and amygdala [25] and improved spatial memory [25,28] without eliciting any detectable inflammatory response [25,26,30]. Likewise, MRI revealed the tendency of scFv-h3D6 treatment to protect against further reduction in brain volume than already existing in 5-month-old 3xTg-AD mice [30]. This observation was corroborated in a longitudinal study where a tendency for protection from the loss of volume associated with AD was found [24]. In addition, magnetic resonance spectroscopy (MRS) quantified the relative concentrations of several AD-related metabolites and showed that scFv-h3D6 treatment decreases mIns levels, a marker of inflammation. A similar decrease in inflammation markers has been observed at the late stages of the disease in the same mouse model [22]. Primary human astrocyte cultures showed the anti-inflammatory properties of scFv-h3D6, as well as served as a model to demonstrate that the effects of scFv–h3D6 and bapineuzumab (mAb-h3D6) are similar [31,32]. Unfortunately, the attempt to compare scFv-h3D6 with bapineuzumab (mAb-m3D6) in terms of inducing ARIA in the 3xTg-AD mouse failed because this model does not develop CAA [33,34]. Therefore, to extend our previous work in the 3xTg-AD mouse model, we decided to use a different transgenic model carrying the same APP mutation (APP_Swe_) but which was reported to suffer from CAA.

Here, the effects of scFv-h3D6 and bapineuzumab (mAb-m3D6) treatments in aged APP23 mice are compared. The APP23 mouse model of AD also constitutes a good model for CAA since ARIA-H appears because of aging [35]. Apart from being effective in reducing Aβ to the same extent as the complete antibody, scFv-h3D6 prevented induced hemorrhagic events. Although the reduction in Aβ levels is not enough to ameliorate AD neurodegeneration in the late stage of the disease, this work is the first one showing that the use of scFvs could be a good approach to prevent the main severe side-effects of Aβ-immunotherapy with full-length antibodies.

## 2. Materials and Methods

### 2.1. ScFv-h3D6 Production

ScFv-h3D6 was produced as previously described [26]. Briefly, protein expression was carried out using the pET28a (+) vector and *E. coli* BL21 (DE3) strain. Induction with 0.5 mM IPTG (isopropyl β-D-thiogalactopyranoside) was performed at OD_600_ = 0.7 and incubation at 37 ˚C for 18 h. After three freeze–thaw cycles, the cellular pellet was sonicated for 5 min, at 70% duty cycle and output 9 (Sonifier 450, Branson, MO, USA). After a centrifugation step at 100,000× *g*, 4 ˚C for 1 h, the insoluble fraction was dissolved in denaturing buffer (100 mM Tris-HCl, 10 mM GSH, pH 8.5, and 8 M urea) and refolded by dilution (1:10) in ice-cold refolding buffer (100 mM Tris/HCl, 100 mM L-arginine, and 0.15 mM GSSG, pH 8.5) for 48 h. Then, a cationic exchange chromatography (Resource S6, GE Healthcare, Chicago, IL, USA) using 5 mM Na_2_HPO_4_, pH 6.5 buffer, and a gradient up to 15% of the same buffer plus 1 M NaCl was performed. Lipopolysaccharides were removed from the protein by using Detoxi-Gel Endotoxin Removing columns (Thermo Scientific, Waltham, MA, USA). The buffer was changed to PBS (pH 7.4) using PD-10 Desalting Columns (GE) and stored at −20 °C until its use.

### 2.2. mAb-m3D6 Production

mAb-m3D6, the mouse form of bapineuzumab, was expressed by hybridoma clone RB96 3D6.32.2.4 (PTA-5130, ATCC, Manassas, VA, USA) in the Cell Culture, Antibody and Cytometry Facility (Servei de Cultius cel·lulars, producció d’Anticossos i Citometria, SCAC) at the UAB. Briefly, 10 mL of DMEM W/Glutamax (Dulbecco’s Modified Eagle’s Medium, glutamine supplemented, Gibco, Waltham, MA, USA) were added to the initial vial and mixed before cells were separated from the medium by centrifugation at 200× *g*, 4 ˚C for 5 min. Cells were re-suspended in DMEM supplemented with 10% FCS (fetal calf serum, Life Technologies, Carlsbad, CA, USA) and grown up to 1,000,000 cells/mL. During the expansion of the culture in T-75 flasks (ThermoFisher, MA, USA), the medium was changed every 3 days. Once Bioreactor Cell Line 350 (VWR, Radnor, PA, USA) was inoculated, the medium was pooled and changed every 7 days. The number of cells in the bioreactor was kept under 135 M cells. The supernatant pool was purified through protein-A affinity chromatography (HiTrap MAb Select, GE Healthcare, Chicago, IL, USA) in a BioLogic LP (Low-pressure Chromatography System, BioRad, Hercules, CA, USA) after dilution in 20 mM Na2PO4, pH 7.0. Elution was performed in 50 mM Gly buffer (pH 3.0), and the sample was neutralized with 1 M Tris-HCl, pH 9.0. Finally, pure mAb-m3D6 was dialyzed to PBS (pH 7.4) and stored at −20 °C until further use.

### 2.3. Animals

APP23 (B6.Cg-Tg(Thy1-APP)3Somm/J) mice and their genetic background (C57BL/6, hereafter non-transgenic, NTg mice) were purchased from Jackson Laboratories and housed at the animal facility of the Vall d’Hebron Research Institute (Barcelona, Spain). Animals were housed under standard laboratory conditions (22 ± 2 °C, 55 ± 5% of 12 h light/dark cycles) with ad libitum access to food and water under the supervision of the animal facility staff. Eighteen 27-month-old APP23 male mice were randomly distributed into 3 groups (*n* = 6), and 6 age- and gender-matched NTg mice constituted the reference for the non-pathological condition. There are no differences between male and female mice in CAA frequency and severity. Investigators responsible for MRI and histological evaluations were blinded to group allocation throughout the assessment process.

### 2.4. Experimental Design and Statistics

**Figure 1:**
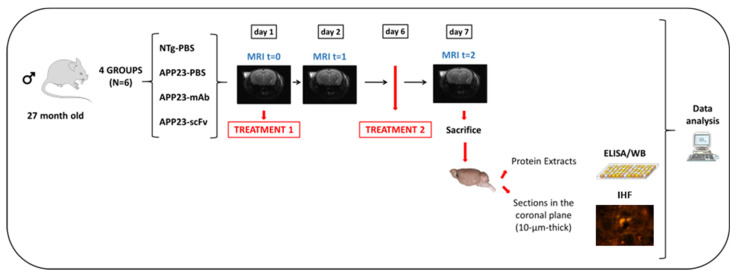
Experimental Design. Four experimental groups were assembled (*n* = 6); one PBS-treated non-transgenic mouse (NTg) group and three groups with 6 randomly assigned 27-month-old male mice each: PBS-treated APP23 mice; mAb-m3D6-treated APP23 mice; and scFv-h3D6-treated APP23 mice. Mice were intraperitoneally injected twice with 200 μL PBS or treatments. The APP23-mAb group received two treatments with mAb-m3D6 (4.5 μg/μL; 12.4 mg/kg). The APP23-scFv group received two treatments with scFv-h3D6 (1.5 μg/μL; 4.1 mg/kg). MRI sessions were performed at the beginning of the experiment, as a baseline, and 24 h after each administration. The second treatment was administered 5 days after the first one. Finally, mice were euthanized, and one hemisphere was processed for biochemical analysis and the other for histological analysis.

Figure 1 shows the overall experimental design. The NTg-PBS and APP23-PBS groups received two intraperitoneally administered 200 μL treatments with PBS each. The APP23-mAb group received two 200 μL treatments with mAb-m3D6 in PBS at a concentration of 4.5 μg/μL, which corresponded to 12.4 mg/kg doses. The APP23-scFv group received two 200 μL treatments with scFv-h3D6 in PBS at a concentration of 1.5 μg/μL, which corresponded to 4.1 mg/kg doses. Such a 1:3 ratio is equimolar in terms of paratopes considering the ratio between the Mw of scFv molecules (25 kDa) and of mAbs (150 kDa), as well as the fact that scFv molecules have 1 paratope and mAbs have 2. Mice were subjected to three brain MRI sessions. The first one before treatment 1, the second one 24 h after treatment 1, and the third one 24 h after treatment 2, which was performed 5 days after treatment 1 (this interval was based on the pharmacokinetics of scFv-h3D6 [29]). Animals were euthanized and brains obtained; one half was processed for histological sections and the other half was frozen in liquid nitrogen to obtain extracts for biochemical testing.

All procedures were performed in accordance with the European guidelines for the care and use of laboratory animals (Directive 2010/63/EU) and were approved by the Ethics Committee on Animal and Human Research at the Universitat Autònoma de Barcelona and by the Catalan Government (CEEAH 0661).

Because of the difficulty in obtaining sex-matched aged animals, the sample size per group was small (*n* = 6). Therefore, statistical differences among groups were evaluated with the non-parametric Kruskal–Wallis test. Next, a two-to-two comparison was performed using the Mann–Whitney U test. Values were expressed by medians, maximum, and minimum, and depicted as box plots. A *p*-value ≤ 0.05 was considered statistically significant. A *p*-value between 0.051 and 0.100 was considered a tendency [36]. Effect size was measured by computing Rank-biserial correlation coefficients with the use of the Wendt formula [37]:

(1) r = 1 − (2U/n_1_n_2_),

where U is the Mann–Whitney statistics, and n_1_ and n_2_ the sizes of the samples being compared. The correlation r expresses the difference between the proportions of pairs that support the hypothesis minus the proportion of pairs that do not. For n_1_ = 6 and n_2_ = 6, the minimum value of r significant at *p*-value ≤ 0.05 is 0.61 [37].

### 2.5. Magnetic Resonance Imaging (MRI)

Acquisitions were performed at the joint nuclear magnetic resonance facility of the Universitat Autònoma de Barcelona and Centro de Investigación Biomédica en Red—Bioingeniería, Biomateriales y Nanomedicina (CIBER-BBN) (Cerdanyola del Vallès, Spain), Unit 25 of NANBIOSIS. Experiments were conducted on a 7T Bruker BioSpec 70/30USR scanner (Bruker BioSpin GmbH, Ettlingen, Germany) equipped with a 72 mm inner diameter linear volume coil as a transmitter and a dedicated mouse brain surface coil as a receiver.

Mice were anesthetized (2% isoflurane in 1 L/min oxygen) and introduced in the magnet. Animals were provided with anesthesia continuously throughout the acquisition procedure via a nosecone. An animal monitoring and control system (SA Instruments, Stony Brook, NY, USA) was used to control the respiration rate (50–100 bpm) and core body temperature (37 ± 1 °C) was maintained via integrated water hoses in the animal’s bed during the whole session. Data were acquired and processed on a Linux computer using the ParaVision 5.1 software (Bruker BioSpin GmbH, Ettlingen, Germany). Homogenization of the magnetic field was performed for every animal and imaging session before image acquisition.

#### 2.5.1. Volume Measurements

For brain volume measurements, high-resolution T2-weighted (HR T2w) images were acquired using a Multislice Rapid Acquisition with Relaxation Enhancement (RARE) sequence with RARE factor = 8, effective echo time (TE_eff_) = 36 ms, and repetition time (TR) = 4200 ms. Thirty 0.5 mm thick contiguous slices covering the whole mouse brain were acquired using a field of view (FOV) = 1.92 cm × 1.92 cm, matrix size (MTX) = 256 × 256, and number of averages (NA) = 4. ImageJ 1.54 Software (NIH, Maryland, MD, USA) was used to calculate the volume of the different regions as the number of pixels within the brain multiplied by the pixel volume (pixel volume (mm^3^) = [(FOV_X_ (mm) × FOV_Y_ (mm))/(MTX_X_ × MTX_Y_)] × 0.5 mm). Regions of interest (ROIs) were manually selected from sections corresponding to anatomical positions from +3.14 mm to −4.92 mm of Bregma (The Mouse Brain in Stereotaxic Coordinates [38]).

#### 2.5.2. Imaging ARIA-H

To visualize ARIA-H, HR T2*w images covering the whole brain were acquired using the same geometry parameters as in HR T2w images. Images were obtained using a fast low-angle shot (FLASH)-gradient echo sequence with TE/TR = 8 ms/500 ms, flip angle = 40°, and NA = 8. Spherical hypointense signals on T2*w images with a minimum diameter of 150 μm, located in the cortex, hippocampus, and thalamus/striatum, were visually counted as hemorrhagic lesions. However, hypointense signals that follow the anatomical course of arteries or veins are excluded from this count, as they represent normal susceptibility effects from vascular structures rather than parenchymal hemorrhagic lesions. To avoid counting the same hemorrhagic lesion multiple times, lesion presence was carefully controlled across consecutive slices. We have previously reported a correlation between hypointensities detected using a similar protocol and Prussian Blue staining, confirming ferric deposition and thereby validating the identification of hemorrhagic lesions [39]. New lesions were identified by systematically comparing T2*-weighted images slice by slice with those acquired at earlier time points. Analyses were performed by a single trained investigator. Hemorrhagic lesion volume was quantified using ImageJ 1.54 Software (NIH, Maryland, MD, USA) considering the measured area of each lesion multiplied by the slice thickness (500 μm) and normalized by the relative signal loss between lesion signals and the surrounding tissue signal.

#### 2.5.3. Imaging ARIA-E

As mentioned before, FLAIR MRI hyperintensities constituted the first evidence of ARIA-E in a phase 1 clinical trial with bapineuzumab. This MRI sequence is essentially a T2w with suppression of ventricular water. In high-field pre-clinical settings, FLAIR is not commonly used because longer acquisition times are needed, and it is inefficient in totally suppressing ventricular water. Instead, HR T2w images are used to detect the presence of ARIA-E. This information is complemented by apparent diffusion coefficient (*ADC*) determinations indicative as well of ARIA-E. *ADC* data were extracted from diffusion tensor imaging (DTI) acquired using a diffusion-segmented echo planar imaging sequence with respiration gating for minimizing motion artifacts (TE = 28 ms, TR = 2600 ms, FOV = 1.92 cm × 1.92 cm, and MTX = 96 × 96). Ten 1 mm thick contiguous slices were acquired. For each slice, five non-diffusion-weighted (b = 0 s/mm^2^) and diffusion-weighted images in 20 different diffusion gradient directions using 3 b-values (200, 600, and 1000 s/mm^2^) were acquired with diffusion gradient time = 5 ms, and time between diffusion gradients = 15 ms. Diffusion-weighted images were processed with the Paravision 5.1 software to obtain the diffusion tensor providing *ADC* values as the mean of the principal diffusivities (*λ*_1_, *λ*_2_, and *λ*_3_) of the diagonalized matrix [40]. Finally, mean *ADC* values were obtained from ROIs manually selected from the cortex, hippocampus and striatum/thalamus, from sections corresponding to anatomical positions from +3.14 mm to −4.92 mm, from −1.28 mm to −2.75 mm, and from 1.64 mm to −2.75 mm of Bregma (The Mouse Brain in Stereotaxic Coordinates [38]), respectively.

#### 2.5.4. Axonal and Myelin Damage

DTI also allows for the detection of alterations in white matter, and a decrease in fractional anisotropy (*FA*, Equation (2)), specifically, is generally interpreted as an indicator of neurological damage. *FA* quantifies the degree to which water diffusion is directionally constrained by axonal fiber orientation (anisotropy). *FA* values range from 0, representing isotropic diffusion where molecular displacement is equally unrestricted in all directions, to 1, corresponding to perfectly anisotropic diffusion [40]. However, a decrease in *FA* alone does not allow for the discrimination between axonal and myelin degradation. To address this limitation, additional metrics are considered (Equation (3)): axial diffusivity (*λ*_||_), describing water movement parallel to axonal fibers, which is associated with axonal integrity, and radial diffusivity (*λ*_⊥_), representing water movement perpendicular to axonal tracts, which is thought to be a marker of myelin pathology [41].

(2) $$FA=\sqrt{\frac{3\;\left[{\left(\lambda_{1}-ADC\right)}^{2}+{\left(\lambda_{2}-ADC\right)}^{2}+{\left(\lambda_{3}-ADC\right)}^{2}\right]}{2({\lambda_{1}}^{2}+{\lambda_{2}}^{2}+{\lambda_{3}}^{2})}},$$

(3) $$\lambda_{\left|\right|}=\lambda_{1}\;;\;\lambda_{⊥}=\frac{\left(\lambda_{2}-\lambda_{3}\right)}{2},$$

Regions of interest (ROIs) were manually selected from the fractional anisotropy, and each of the principal diffusivity maps, from the rostral, middle, and caudal corpus callosum, from the midline to under the peak of the cingulum. Coordinates corresponded to anatomical positions centered at +1.32 mm, −0.64 mm, and −1.64 mm of Bregma (The Mouse Brain in Stereotaxic Coordinates [38]), respectively, using the point at which the anterior commissure crossed the midline to register alignments.

### 2.6. Sample Collection and Processing

Animals were euthanized by decapitation after the last MRI acquisition. Their brains were immediately removed, rinsed in cold PBS and dissected on ice. One hemisphere was directly frozen and stored at −80 °C until use. After defrosting, the cerebral cortex and hippocampus were dissected and mechanically homogenized in cold TBS buffer (pH 7.6) supplemented with a protease inhibitor cocktail (Roche). Samples were centrifuged at 100,000× *g* at 4 °C for 1 h after sonication (1 cycle of 35 s, at 35% duty cycle, output 4 in a Dyantech Sonic Dismembrator ARTEK 300) (Biologics Inc., Manassas, VA, USA). The supernatant was stored as the TBS-soluble fraction (corresponding to the extracellular soluble fraction) and the pellet was re-suspended in cold TBS supplemented with inhibitor cocktail and 2% of SDS. After sonication and centrifugation, the supernatant was stored as the SDS-soluble fraction (corresponding to the intracellular soluble fraction). Finally, the pellet was re-suspended in 70% formic acid, sonicated, and centrifuged. The supernatant was dried overnight in a vacuum concentrator (Savant SpeedVac, Thermo Fisher Scientific, Waltham, MA, USA). The extract was re-suspended in DMSO and labeled as the FA-soluble fraction (corresponding to the insoluble aggregated fraction). All fractions were kept at −80 °C until use. The hemisphere was fixed by 4% paraformaldehyde (PFA) immersion for 48 h. Then, samples were embedded in paraffin following common procedures, serially sectioned in coronal plane, and mounted on SuperfrostTM Plus microscope slides (Thermo Fisher Scientific).

### 2.7. Immunofluorescence

Samples were deparaffinized, and antigen retrieval and incubation with the corresponding primary antibody (mouse anti-Aβ 6E10, 1:200 Covance Signet, ref. SIG-39320-200, lot. D13BF00601; lab-made anti-scFv-h3D6 polyclonal antibodies [29], 1:100) were performed as described in the previous section. After washing, samples were incubated with the corresponding secondary antibody (goat anti-mouse IgG, Cy3 conjugated, Chemicon, Millipore, ref. AP124C; and goat anti-rabbit IgG, Alexa488 conjugated, Chemicon, Millipore, ref. AP123F) for 1 h at RT and cover-slipped with antifade Vectashield mounting medium (Vector Laboratories, Burlingame, CA, USA).

### 2.8. Image Capture and Processing

A Leica DMRB microscope with a Leica DFC 500 camera was used to capture images. The cerebral sections corresponded to the range of coordinates from Figure 43 (interaural 2.34 mm and Bregma −1.46 mm) to Figure 48 (interaural 1.74 mm and Bregma −2.06 mm) in The Mouse Brain in Stereotaxic Coordinates [38]. The ImageJ software was used for images analysis.

### 2.9. Enzyme-Linked Immunosorbent Assays (ELISAs)

Aβ_40_, Aβ_42_, TNFα, IL-6, IL-1β, and IL-33 levels were quantified by ELISA following the protocol recommended by each manufacturer (Human Aβ_40_ ELISA kit, Invitrogen, Carlsbad, CA, USA, ref. KHB3481; Human Aβ_42_ ELISA kit, Invitrogen, ref. KHB3441; Mouse Duoset TNFα ELISA, RD Systems, ref.DY410-05; Mouse Duoset IL-6 ELISA, RD Systems, ref. DY401-05; Mouse Duoset IL-1β ELISA, RD Systems, ref. DY406-05; and Mouse Duoset IL-33 ELISA, RD Systems, ref. DY3626-05). Data were normalized to the total amount of protein in each sample (Pierce BCA Protein Assay Kit, Thermo Scientific, ref. 23225).

## 3. Results

Because we already demonstrated that the effects of scFv-h3D6 and bapineuzumab (mAb-h3D6) are similar in human primary astrocyte cultures [31,32], we first focus on security issues and then validate that efficacy is also similar when comparing scFv–h3D6 and mAb-m3D6 treatments in APP23 mice.

### 3.1. Hemorrhagic Events Induced by mAb-m3D6 Are Prevented by scFv-h3D6

**Figure 2:**
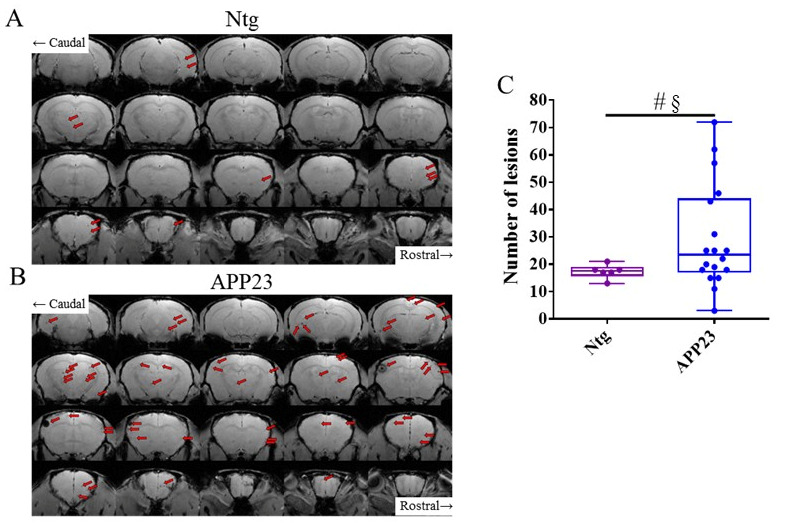
T_2_*w images showing cerebral microhemorrhages prior to treatments. Representative coronal sections of the brains of NTg (**A**) and APP23 (**B**) mice. Microhemorrhages, visualized as spherical hypointense signals within the brain parenchyma, are indicated by red arrows. (**C**) Total number of cerebral lesions in NTg (purple) and APP23 (blue) mice prior to treatments. Data are expressed as medians in box plots, and whiskers represent the minimum and maximum values. Statistical differences were assessed with the non-parametrical Mann–Whitney U test. # Tendency *p* ≤ 0.1, and § r ≥ 0.61.

Table 1 compiles the data for the number of microhemorrhages in different parts of the brain in the different experimental groups in the study, as detected by T_2_*w MRI [35,42]. Aged NTg (C57BL/6) mice showed some hypointense lesions, associated with microhemorrhages, in different parts of the brain (Figure 2A, attributable to normal aging). APP23 mice showed a higher number of microhemorrhages than NTg mice (Figure 2A,B). Hypointense lesions in the APP23 brain were mainly detectable in the cortex and thalamus/striatum (Figure S1). In agreement with these results, the quantification of the number of lesions in NTg and APP23 mice prior to treatments shows that APP23 tends to display a higher number of microhemorrhages (Figure 2C), as expected for a CAA model (U = 28, *p* = 0.085). Effect size could not be applied to solve the meaning of this tendency because the sizes of the groups were too different (*n*, Ntg = 6; *n*, APP23 = 18).

**Figure 3:**
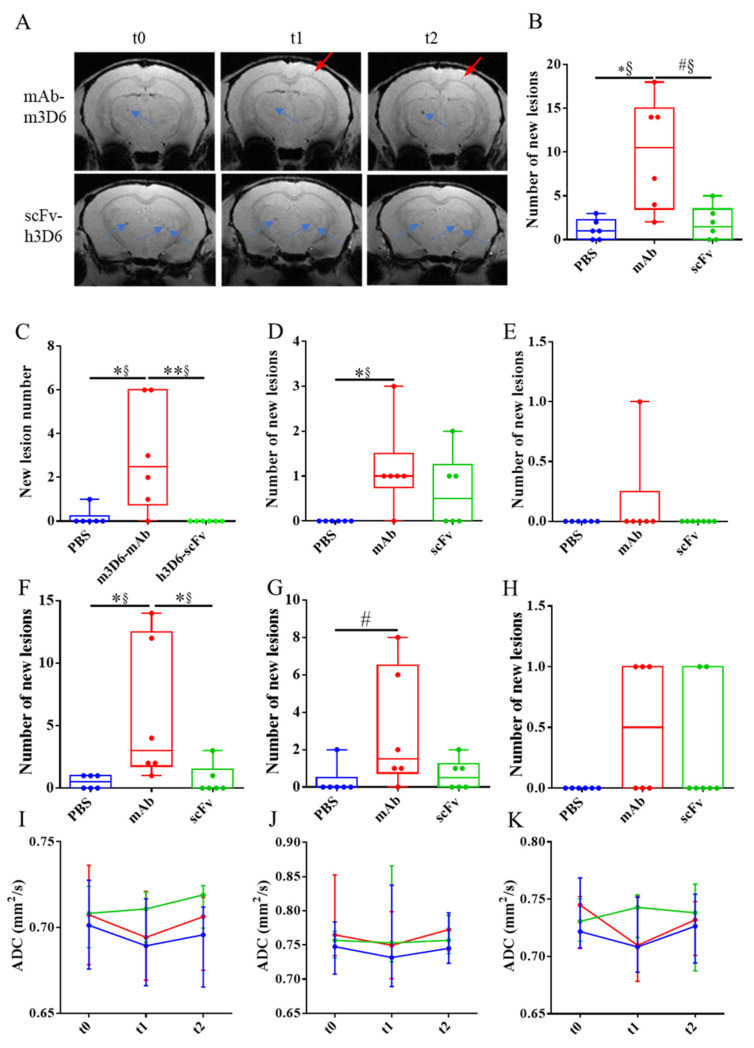
T_2_*w images showing cerebral microhemorrhages during the experiment. (**A**) Representative coronal brain section of APP23 mice treated with mAb-m3D6 (**top**) at the beginning of the experiment (t0, **left**), after treatment 1 (**center**), and after treatment 2 (**right**); and representative coronal brain section of APP23 mice treated with scFv-h3D6 (**bottom**) at the same times. Lesions a t = 0, blue arrows and new lesions, red arrows. (**B**) Number of new lesions induced by PBS (blue), mAb-m3D6 (red), and scFv-h3D6 (green) at the end of the study. (**C**,**D**) New lesions after the first administration in the cortex (**C**), striatum/thalamus (**D**), and hippocampus (**E**). (**F**–**H**) New lesions after the second administration in the cortex (**F**), striatum/thalamus (**G**), and hippocampus (**H**). (**I**–**K**) Temporal evolution of the apparent diffusion coefficient (*ADC*) values induced by PBS (blue), mAb-m3D6 (red), and scFv-h3D6 (green) in the cortex (**I**), striatum/thalamus (**J**), and hippocampus (**K**). No significant differences were observed in *ADC* values. An increase in *ADC* would typically be expected in the presence of vasogenic edema. Data are expressed as medians in box plots, and whiskers represent the minimum and maximum values. Statistical differences were assessed with the non-parametrical Mann–Whitney U test. ** Significant differences *p* ≤ 0.01; * Significant differences *p* ≤ 0.05; # Tendency *p* ≤ 0.1; and § r ≥ 0.61.

The main adverse effect of Aβ-immunotherapy for AD is the development of ARIA-E and ARIA-H through a mechanism that involves Fc-mediated microglial activation and subsequent inflammation [17,18]. To test this hypothesis, we previously designed and produced an antibody fragment lacking the Fc region from bapineuzumab. Compared with the PBS-treated group, the full-length antibody mAb-m3D6 produced more new hemorrhagic events (U = 1.5, *p* = 0.0174, r = 0.92) than scFv-h3D6 (U = 16.5, *p* > 0.9999, r = 0.08) at the end of the treatments (Figure 3A,B). Specifically, the mAb-m3D6 treated group showed a significant increase in new microhemorrhages in the cortex (U = 1.5, *p* = 0.0376, r = 0.92) and a tendency to increase the number of new lesions in the thalamus/striatum (U = 6, *p* = 0.0813, r = 0.67) (Figure 3F–H). Indeed, mAb-m3D6 induced new lesions in the cortex (U = 4, *p* = 0.0297, r = 0.77) and the thalamus/striatum (U = 3, *p* = 0.0208, r = 0.83) after a single dose; whereas the scFv-h3D6-treated group behaved as the PBS group did (Figure 3C–E). It is worth noting that treatments did not induce an increase in the volume of the lesions, as compared with the sporadic increase in volume observed in the PBS-treated group (Figure S2).

Concerning ARIA-E, no differences were detected based on *ADC* parameter determinations (Figure 3I–K). An increase in *ADC* would typically be expected in the presence of vasogenic edema.

### 3.2. Both, mAb-m3D6 and scFv-h3D6 Do Not Trigger a General Inflammatory Response

Although it is not clear whether the immune system activation is a cause or consequence of AD, it is widely accepted that it plays a pivotal role in the progression of the disease [43]. We have previously demonstrated that scFv-h3D6 does not produce any detectable inflammatory response in 3xTg-AD mice [22,25,26,30]. Here, the effect of the treatments on inflammation in APP23 mice is shown.

Although neuroinflammation is a characteristic of AD, 27-month-old mice are so advanced in age that it could be plausible that no big differences between WT and APP23 would be evident. Moreover, it will be interesting to analyze whether treatments induce an inflammatory response or not.

**Figure 4:**
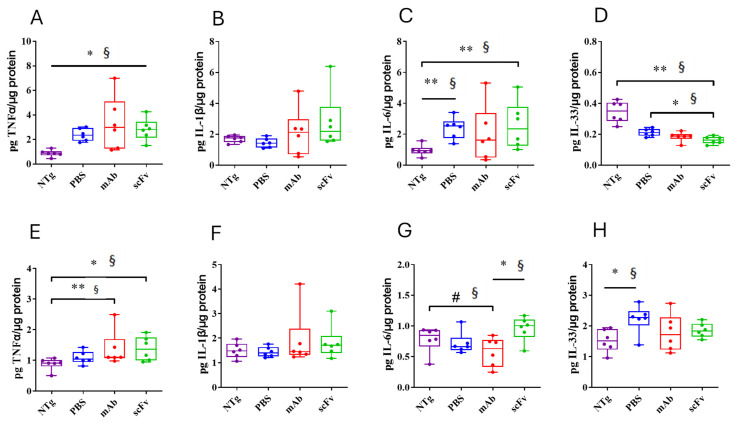
Effect of mAb-m3D6 and scFv-h3D6 treatments in the inflammatory response in the hippocampus and in the cortex. Interleukin levels were measured by ELISA and normalized by the total amount of protein in the TBS-soluble fraction. (**A**–**D**) Hippocampus TNF-α (**A**), IL-33 (**B**), IL-1β (**C**), and IL-6 (**D**) levels. (**E**–**H**) **Cortex** TNF-α (**E**), IL-33 (**F**), IL-1β (**G**), and IL-6 (**H**) levels. Data is expressed as medians in box plots, and whiskers represent the minimum and maximum values. Statistical differences were assessed with the non-parametrical Mann–Whitney U test. ** Significant differences *p* < 0.01, * Significant differences *p* < 0.05, # Tendency *p* < 0.1, and § r ≥ 0.61.

For an in-depth analysis of the inflammatory state of the untreated and treated APP23 mice, the levels of TNF-α, IL1-β, IL-6, and IL-33 in the TBS-soluble fraction of the hippocampus (Figure 4A–D) and cortex (Figure 4E-H) have been quantified. An evident proinflammatory state in the hippocampus of APP23 mice was observed. An increase in TNFα (U = 0; *p* = 0.0022; r = 1) and IL-6 (U = 1; *p* = 0.0043; r = 0.94), and a reduction in the anti-inflammatory IL-33 [44] (U = 0; *p* = 0.0022; r = 1) levels were found. Although both m3D6 and scFv-h3D6 failed in reversing these imbalances in the hippocampus of APP23 mice, probably because of the advanced stage of the disease and the short period of the treatments, it is worth noting that a general exacerbated immune response was not produced after treatments. In contrast to the hippocampus, no differences in interleukin levels between WT and APP23 mice were found in the cortex. However, a slight increase in TNFα levels after mAb-m3D6 (U = 2; *p* = 0.0087; r = 0.88) and scFv-h3D6 (U = 3; *p* = 0.0152; r = 0.83) administration with respect to the untreated WT mice was observed. IL-6, the expression of which was enhanced in astrocytes culture [32] by scFv-h3D6 but not by mAb-m3D6, was also slightly increased in the scFv-h3D6-treated mice relative to the mAb-m3D6-treated APP23 mice (U = 3; *p* = 0.0152; r = 0.83). This slight difference could be due to the different mechanisms of clearance of the scFv-Aβ complex with respect to the Fc-mediated clearance of the mAb-Aβ complex. In addition, these results suggest that both the mAb-m3D6 and the scFv-h3D6 do not trigger a general neuroinflammatory process.

### 3.3. Both mAb-m3D6 and scFv-h3D6 Reduce Aβ Levels

The full-length antibody bapineuzumab reduced Aβ in humans [45], even though no clinical improvement was achieved. We have previously reported that scFv-h3D6 reduced both intracellular and extracellular Aβ levels in young and aged 3xTg-AD mice without eliciting any detectable inflammatory response [22,25,26,30]. Here, the comparative effects of mAb-m3D6 and scFv-h3D6 on Aβ clearance are shown.

It has been reported that anti-Aβ antibodies are accumulated in the periventricular areas because of the difficulty in crossing the blood–brain barrier (BBB) [46]. In fact, we previously showed that after 2 weeks of treatment, scFv-h3D6 co-localizes with intracellular (SDS fraction) and also extracellular Aβ (TBS fraction) in the hippocampus but not in the cortex of 22-month-old 3xTg-AD mice [22].

**Figure 5:**
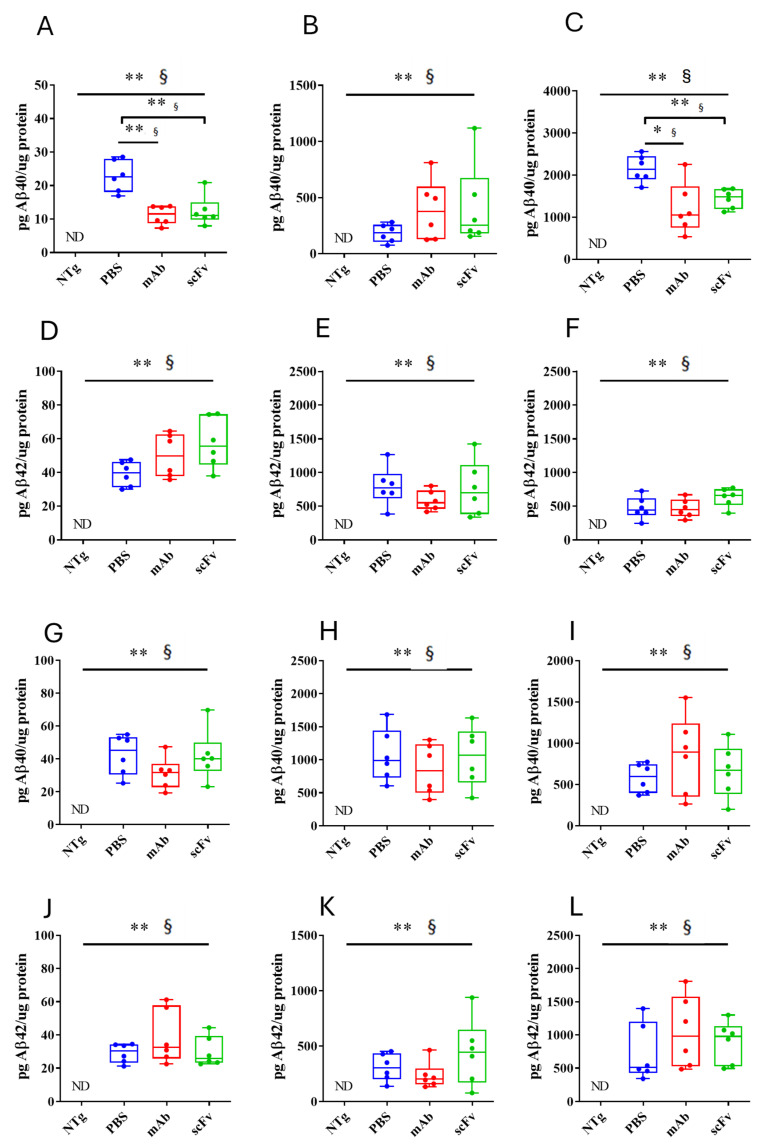
Effect of mAb-m3D6 and scFv-h3D6 treatment in Aβ burden. Aβ levels as quantified by ELISA and normalized by the total amount of protein. Hippocampus (**A**–**F**) Aβ_40_ levels in the TBS-soluble fraction (**A**), SDS-soluble fraction (**B**), and *FA*-soluble (**C**) fraction. Aβ_42_ levels in the TBS-soluble fraction (**D**), SDS-soluble fraction (**E**), and *FA*-soluble fraction (**F**). Cortex (**G**–**L**) Aβ_40_ levels in the TBS-soluble fraction (**G**), SDS-soluble fraction (**H**), and *FA*-soluble (**I**) fraction. Aβ_42_ levels in the TBS-soluble fraction (**J**), SDS-soluble fraction (**K**), and *FA*-soluble fraction (**L**). Data is expressed as medians in box plots, and whiskers represent the minimum and maximum values. Statistical differences were assessed with the non-parametrical Mann–Whitney U test. ** Significant differences *p* < 0.01, * Significant differences *p* < 0.05, and § r ≥ 0.61.

The quantification of Aβ_40_ and Aβ_42_ levels by ELISA in the APP23 mice’s hippocampus (Figure 5A–F) and cortex (Figure 5G–L) showed high levels in the PBS-treated animals. Aβ_40_ levels were higher in the SDS- and FA-soluble fractions than in the TBS fraction, indicating the predominance of the aggregated form of Aβ at this advanced stage of the pathology. Concerning treatments, mAb-m3D6 and scFv-h3D6 reduced Aβ_40_ levels in TBS-soluble (U = 0, *p* = 0.0022, r = 1 and U = 2; *p* = 0.0087, r = 0.88, respectively) and FA-soluble (U = 3, *p* = 0.0152, r = 0.83 and U = 0, *p* = 0.0022, r = 1, respectively) fractions in the hippocampus (Figure 5A–C). However, treatments were not effective against Aβ_42_. This concurs with the above-mentioned fact that bapineuzumab was designed against the first five residues in the N-terminus region of the Aβ peptide. Aβ_40_ aggregates in multiples of 3 (trimers, hexamers, and nonamers, etc.), giving rise to a fibril where the N-terminus of the three peptides is exposed to the solvent in the vertex of each trimer (PDB 2M4J) [9]. In contrast, Aβ_42_ aggregates as dimers partially burying the N-terminus of the peptides in the core of the Aβ_42_ fibril (PDB 5OQV) [10]. Therefore, bapineuzumab-based treatments were effective against Aβ_40_ but not Aβ_42_ aggregates. Although both treatments reduced Aβ levels, the difficulty of crossing the BBB and the advanced stage of AD in the aged APP23 mice supports the accepted idea of Aβ-immunotherapy being more effective at the early stages of the disease. Predictably, both the mAb-m3D6 and the scFv-h3D6 colocalized with the mouse Aβ peptide in the hippocampus, as detected by immunofluorescence with 6E10 mab (Figure S3).

As already observed in 22-month-old 3xTg-AD mice [22], no differences in either Aβ_40_ or Aβ_42_ levels among the non-treated, mAb-m3D6-treated and scFv-h3D6-treated APP23 mice were found in the cortex (Figure 5G–F).

### 3.4. Reduction in Aβ Is Not Enough to Recover from Neurodegeneration in Late Stages of AD

Brain atrophy is one of the anatomical hallmarks of AD, so high-resolution T_2_-weighted images are a useful technique for the quantification of brain volume as a therapeutic effect reporter. In previous studies in 3xTg-AD mice at 5 and 12 months of age, cerebral volume was reported to be smaller than in NTg mice [24,30]. Interestingly, such a reduced cerebral volume was partially recovered after scFv-h3D6 treatment.

**Figure 6:**
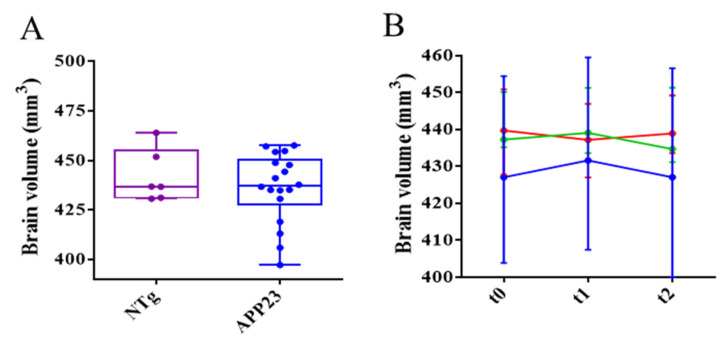
Brain volume quantification from HR T2w images (**A**) Differences in the brain’s volume of NTg (purple) and APP23 (blue) mice at the beginning of the experiment. (**B**) Temporal evolution of brain volume after PBS (blue), mAb-m3D6 (red), and scFv-h3D6 (green) treatments. Data are expressed as medians in box plots, and whiskers represent the minimum and maximum values. Statistical differences were assessed with the non-parametrical Mann–Whitney U test.

No significant differences were observed between 27-month-old NTg and APP23 mice in the cerebral volume (Figure 6A). It is well known that normal aging also produces brain atrophy [47], so this result could be explained by the advanced age of the mice in this study. Neither mAb-m3D6 nor scFv-h3D6 produced changes in the cerebral volume (Figure 6B). This is in contrast with the relation found between neural demise and intracellular Aβ content in different brain areas of 5-month-old 3xTg-AD mice [48]. Interestingly, scFv-h3D6 treatment protected mice from such neurodegeneration at an early stage of the disease [25].

Despite the fact that AD has been described as a gray matter disease, several directional diffusivity studies have revealed the implication of white matter in AD [49,50]. In addition, white matter impairment in the temporal, parietal, and frontal regions, as well as in the corpus callosum, have been associated with problems in language and spatial functions [51]. One of the consequences of AD is axonal damage and myelin impairment, which can be quantified by fractional anisotropy, axial diffusivity, and radial diffusivity [52]. A significant reduction in FA and axial diffusivity, along with a significant increase in radial diffusivity, has been observed in the corpus callosum of 3xTg-AD mice compared to control littermates as early as at 2 months of age [53]. In a study with early AD patients, who already have mild cognitive impairment, an increase in radial diffusivity, associated with demyelinating damage, was detected, as well as a decrease in axial diffusivity, associated with axonal injury, in cortical regions. In groups with high AD risks, similar results indicating that the white matter’s structural integrity was compromised were obtained [54,55].

**Figure 7:**
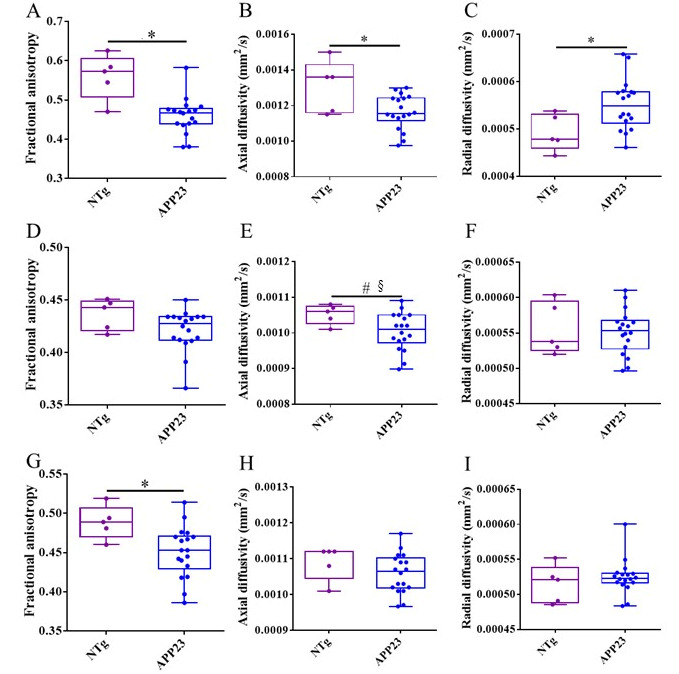
Differences in axonal and myelin integrity between NTg and APP23 mice assessed by DTI across rostrocaudal sections of the corpus callosum (CC). (**A**–**C**) Quantification of fractional anisotropy (*FA*) (**A**), and axial (**B**) and radial (**C**) diffusivity values in the rostral CC of NTg (purple) and APP23 (blue) mice. (**D**–**F**) Quantification of *FA* (**D**), and axial (**E**) and radial (**F**) diffusivity values in the middle CC of NTg and APP23 mice. (**G**–**I**) Quantification of *FA* (**G**), and axial (**H**) and radial (**I**) diffusivity values in the caudal CC of NTg and APP23 mice. Data are expressed as medians in box plots, and whiskers represent the minimum and maximum values. Statistical differences were assessed with the non-parametrical Mann–Whitney U test. * Significant differences *p* ≤ 0.05; # Tendency *p* ≤ 0.1, and § r ≥ 0.61.

**Figure 8:**
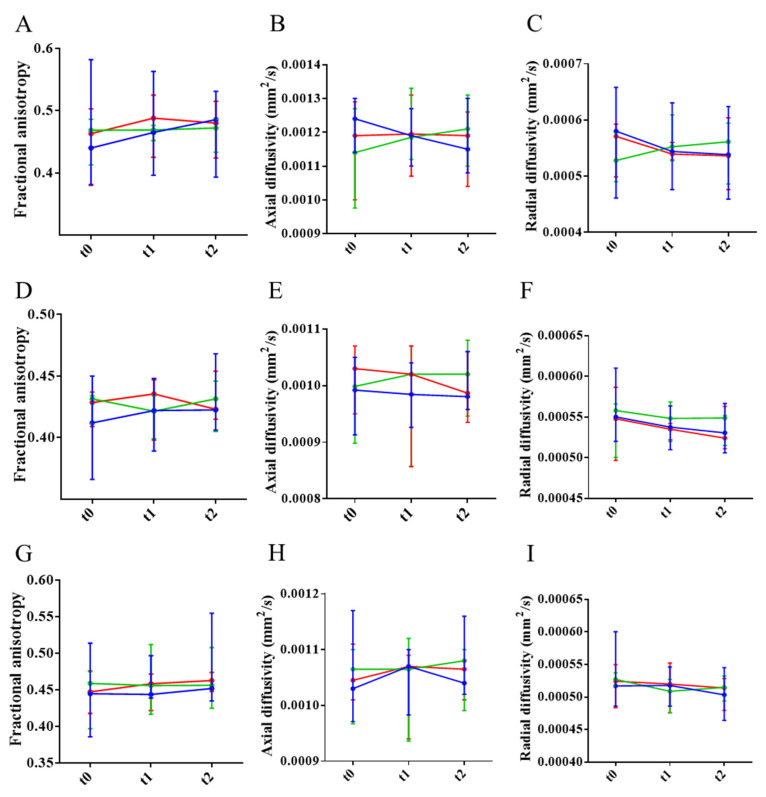
Effect of the treatments in axonal and myelin integrity assessed by DTI evolution across rostrocaudal sections of the corpus callosum (CC). (**A**–**C**) Temporal evolution of fractional anisotropy (*FA*) (**A**), and axial (**B**) and radial (**C**) diffusivity values in the rostral CC of mice treated with PBS (blue), mAb-m3D6 (red), or scFv-h3D6 (green). (**D**–**F**) Temporal evolution of *FA* (**D**), and axial (**E**) and radial (**F**) diffusivity values in the middle CC of mice treated with PBS, mAb-m3D6, or scFv-h3D6. (**G**–**I**) Temporal evolution of *FA* (**G**), and axial (**H**) and radial (**I**) diffusivity values in the caudal CC of mice treated with PBS, mAb-m3D6, or scFv-h3D6. Data are expressed as medians in box plots, and whiskers represent the minimum and maximum values. Statistical differences were assessed with the non-parametrical Mann–Whitney U test.

To assess axonal and myelin impairment, fractional anisotropy (FA) and axial and radial diffusivities were quantified in the frontal, middle, and rostral regions of the CC of NTg and untreated and treated APP23 mice. Results showed that FA and axial diffusivity decreased in the frontal CC, indicating axonal damage. Similarly, radial diffusivity increased, pointing to myelin impairment (Figure 7A–C). In the middle region, only a reduction in axial diffusivity was detected, pointing to some extent of axonal damage (Figure 7D–F). In the rostral region, only a reduction in FA was detected (Figure 7G–I). Therefore, and although all the CC is affected to some extent, the frontal CC is the more affected region. Treatments could not ameliorate axonal or myelin impartment at this advanced stage of the disease (Figure 8).

## 4. Discussion

Therapeutic antibodies against Aβ might not only accelerate vascular deposition but also bind to accessible vascular Aβ and consequently further disrupt vessel integrity, contributing to the leakage of red blood cells (ARIA-H).

To our knowledge, this works is the first one showing that the use of scFvs could be a good approach to prevent the main severe side effects of Aβ-immunotherapy with full-length antibodies. The only relevant work we have found where reduction of ARIA-like lesions has been achieved in a new mouse model, the 5xFAD;TfRmu/hu KI, has just been published and it is focused on a very different construct [56,57]. Specifically, antibody transport vehicle (ATV) with asymmetrical Fc mutations (ATVcisLALA) mediates CNS delivery, enhances brain exposure, biodistribution, and Aβ plaque target engagement throughout the parenchyma. Additional two constructs, ch3D6kLALA (with asymmetric LALA mutations on huIgG Fc) and ATVWT:3D6 (with wild-type huIgG) showed no ARIA by MRI.

In an MRI study of APP-overexpressing mice that had been treated with a mouse analog of bapineuzumab, transient BBB leakage was associated with microbleeds during early treatment, recapitulating some features of ARIA such as the frequent co-occurrence of ARIA-E and ARIA-H; this effect was not seen in saline-treated transgenic mice or immunotherapy-treated wild-type mice [58,59].

The main limitation of this study is that we specifically selected very aged animals (27-months-old), which are at the upper end of the lifespan for this strain (typically 26–30 months). The fragility of animals at this advanced age, combined with the need for sex-matching, restricted the number of APP23 mice available to eighteen. Despite the modest sample size, our statistical analyses revealed significant differences in several key parameters like in the number of microhemorrhages, in the TNF-α, IL-6, and IL-33 levels, or Aβ_40_ levels, among others, underscoring the biological relevance and robustness of our findings.

It is likely that scFv-h3D6 slightly increases interleukin levels in the hippocampus. In previous works, IL-6 levels were also increased by scFv-h3D6 but not mAb-3D6 in astrocyte cultures [32]. However, in 3xTg-AD mice, interleukin levels were slightly reduced after scFv-h3D6 administration [22]. It would be plausible that we are here appreciating the first effects of scFv-h3D6 immunization, which could involve some kind of micro/astroglia re-activation at this late stage of the disease.

Aβ_40_ reduction in this work was not enough to restore neurodegeneration featuring in very-late stages of the disease. Treatments should be started earlier because Aβ acts as a trigger but not as an effector, at least not as a unique effector. In fact, there is a temporal evolution, where increased Aβ deposition precedes hypometabolism that in turn is followed by cortical thinning in humans, as assessed by longitudinally following partial patterns of neuroimaging biomarker changes in individuals with familial AD. The study comprises a wide period, with an estimated years-to-onset ranging from 25 years before the onset, to 10 years after the onset. In addition, huge groups of patients were followed (the analyses included 346 individuals with ^11^C-PIB PET Aβ data, 352 with ^18^F-FDG PET metabolism data, and 377 with MRI volumetric data) [5].

In conclusion, bapineuzumab induces hemorrhagic events in the APP23 mouse model of AD, whereas the derived single-chain variable fragment lacking the Fc region does not. This finding corroborates the hypothesis that the Fc region of antibodies shows effector functions driving to ARIA and proposes the development of antibody formats lacking the Fc effector region for being further tested in clinical trials for AD and other neurodegenerative diseases. This could also be applied to the recently FDA- and EMA-approved antibodies, which unfortunately have been reported to induce ARIAs to an even higher extent than bapineuzumab. In recent clinical trials with other anti-N-terminal-Aβ-targeted mAbs, such as gantenerumab (Hoffmann-La Roche, Basel, Switzerland) and aducanumab (Biogen, MA, USA, terminated in 2024), the incidence of ARIA-E is dose-dependent and *APOEε4*-dependent as well [60,61]. It is important to note that, although with a lower incidence than ARIA-E, ARIA-H also occurs in clinical trials for Aβ-immunotherapy [60,61,62]. In the phase 3 clinical trials of aducanumab, the *APOEε4* allele was associated with higher rates of co-existing ARIA-H and ARIA-E (up to 35% at the highest dose) [60]. Although the incidence of ARIA is higher in the aducanumab trials than in those with bapineuzumab, the decision on the accelerated approval of aducanumab (Aduhelm, Biogen), a fully human mAb targeting the-N-termini (3-6) and mid-region, binding all aggregated forms, was made by the FDA in 2021 [63]. This accelerated approval was controversial because an external FDA advisory committee voted against the approval of the drug and the EMA never approved it. Apart from the occurrence of ARIA, the controversy arose from one phase 3 trial showing cognitive impairment improvement (EMERGE) while another trial does not (ENGAGE) [64]. Finally, in 2024, Biogen decided to discontinue aducanumab development and marketing worldwide, including the completion of a related clinical study [65].

The second disease-modifying anti-Aβ that was FDA-approved was lecanemab (Leqembi, Biogen, MA, USA) (2023), a humanized mAb against the E22G “arctic” mutation, targeting protofibrils. This mAb was found to be effective in reducing Aβ accumulation and slowing down cognitive decline. However, ARIA-E occurred in approximately 10% of the overall population and 14.3% of *APOEε4* carriers [66]. ARIA-E and ARIA-H occurrences were 12.6% (symptomatic 2.8%) and 17.3% (Symptomatic 0.7%) [67]. Two years after FDA approval (April 2025), the EMA approved lecanemab (Leqembi) for the treatment of early-stage AD or mild cognitive impairment, but it can only be administered to people with one or no copies of the *APOEε4* allele.

The latest FDA-approved mAb is a humanized mAb, donanemab (Kisunla, Eli Lilly and Co, IL, USA), which recognizes aa 3–13 and targets the N-terminal pyroglutamate of Aβ [68,69]. The incidences of ARIA-E and ARIA-H in the donanemab group were 24.0% and 26.8%, respectively [70]. More phase 3 clinical trials are being conducted. For a review of FDA-approved mAbs against AD, please see [71]. Concerning approval in Europe, the EMA initially issued a negative opinion. However, the pharmaceutical company has recently requested a re-evaluation, and the EMA accepted. EMA’s current refusal does not mean the end in Europe for donanemab, as the same thing happened in the approval process for lecanemab. However, it is evident that ARIA remains a problem for the treatment of AD with full-length mAb. Therefore, the contribution of this work, showing that antibody fragments lacking the Fc effector region appear safer than full-length antibodies because they do not induce ARIA, opens an exciting window in the search of an effective and safe drug for Alzheimer’s disease. In addition, the use of other antibody fragment formats, including bispecific antibodies, could lead to encouraging combined anti-Aβ and anti-tau therapies.

## 5. Conclusions

Our findings show that bapineuzumab induces hemorrhagic events in the APP23 mouse model of AD, whereas the derived single-chain variable fragment lacking the Fc region does not. This finding corroborates the hypothesis that the Fc region of antibodies shows effector functions driving to ARIA. Therefore, this work proposes to conduct the development of antibody formats lacking the Fc effector region to eventually be tested in clinical trials for AD and other neurodegenerative diseases. This finding is highly relevant in light of the new FDA- and EMA-approved mAbs, the first disease-modifying treatments for AD. Unfortunately, all these approvals include limitations, especially in the case of patients who are diploid for the *APOEε4* allele, who cannot benefit from these treatments because of the occurrence of hemorrhages.

## Figures and Tables

**Table 1:** Number of ARIA-H lesions in HR T2*w images. Twenty-five 0.5 mm thick slices all though the brain (interslice distance of 0.1 mm) were used for counting the number of ARIA-H lesions. Lesions with a minimum diameter of 150 μm were considered. Before the treatments, APP23 showed a higher number of hemorrhages than NTg mice, specifically in the cortex. Effect size analysis was not applied in this case because the *n* of the groups was imbalanced. The number of lesions was not different between treatments, but when the number of new lesions is compared, mAb-m3D6 promoted hemorrhagic events and scFv-h3D6 did not, as effect size analysis confirmed. In the cortex, mAb-m3D6 causes a greater number of lesions after the first administration. Finally, the scFv-h3D6-treated group has more ARIA-H in other regions of the brain at t1; however, this was because the number was already higher in this group at the beginning of the experiment and this has no relevance since there were no new lesions. Cx, cortex; Thal, thalamus/striatum; Hc, hippocampus; and Oth, other regions.

		Ntg (*n* = 6)		APP23 (*n* = 18)		APP23-PBS (*n* = 6)				APP23-mAb-m3D6 (*n* = 6)				APP23-scFv-h3D6 (*n* = 6)			
		Number of Lesions				Number of Lesions		Number of New Lesions		Number of Lesions		Number of New Lesions		Number of Lesions		Number of New Lesions	
		Mean	SD	Mean	SD	Mean	SD	Mean	SD	Mean	SD	Mean	SD	Mean	SD	Mean	SD
Total	t0	17.3	±2.6	**24.9** **#**	±15.9	16.3	±8.4			27.3	±13.0			31.2	±21.0		
	t1					17.0	±9.0	0.7	±1.2	31.8	±17.7	4.5 *§	±2.8	32.0	±20.9	0.8	±1.0
	t2					17.7	±8.8	0.7	±0.8	37.2	±20.5	**5.3 #§**	±0.4	33.0	±21.8	1.0	±1.1
Cx	t0	3.5	±2.8	11.5 *	±10.0	6.5	± 4.8			13.0	±12.1			15.0	±10.9		
	t1					6.7	±4.8	0.2	±0.4	16.2	±14.1	3.2 *§	±2.8	15.0	±10.9	0.0	±0.0
	t2					7.2	±4.6	0.5	±0.6	19.0	±16.7	2.8	±3.4	15.7	±12.0	0.7	±1.2
Thal	t0	10.3	±3.2	10.2	±6.5	8.2	±4.9			9.2	±4.5			13.2	±9.0		
	t1					8.5	±5.4	0.3	±0.8	10.3	±5.4	1.2	±0.10	13.8	±9.3	0.7	±0.8
	t2					8.5	±5.4	0.0	±0.0	12.2	±7.2	**1.8 #**	±2.5	13.8	±9.3	0.0	±0.0
Hc	t0	1.0	±1.1	2.2	±2.0	1.7	±1.8			3.2	±2.7			1.7	±1.4		
	t1					1.7	±1.8	0.0	±0.0	3.3	±2.8	0.2	±0.4	1.7	±1.4	0.0	±0.0
	t2					1.7	±1.8	0.0	±0.0	3.7	±3.0	0.3	±0.5	2.0	±0.9	0.3	±0.5
Oth	t0	2.5	±3.1	1.1	±1.9	0.0	±0.0			2.0	±3.0			1.3	±1.0		
	t1					0.2	±0.4	0.2	±0.4	2.0	±3.0	0.0	±0.0	1.5 *§	±1.0	0.2	±0.4
	t2					0.3	±0.8	0.2	±0.4	2.3	± 3.3	0.3	±0.5	1.5	±1.0	0.0	±0.0

Statistical differences were assessed with the non-parametrical Mann–Whitney U test comparing treatments with the APP23-PBS control group. * Significant differences *p* ≤ 0.05; # Tendency *p* ≤ 0.1; and § r ≥ 0.61.

## Data Availability

Data sets generated during and/or analyzed during the current study are available from the corresponding author on reasonable request.

## Supplementary Materials

The following supporting information can be downloaded at https://www.mdpi.com/article/10.3390/biom15111602/s1. Figure S1: Quantification of the number of cerebral microhemorrhages from T2*w images in different brain regions prior to treatments; Figure S2: Determination of the volume of existing cerebral microhemorrhages from T2*w images after treatments. Figure S3: Colocalization of mAb-m3D6 and scFv-h3D6 with the Aβ peptide in the hippocampus.

## Abbreviations

The following abbreviations are used in this manuscript:

Aβ	β-amyloid
AD	Alzheimer’s disease
ADC	Apparent diffusion coefficient
ARIA	Amyloid-related imaging abnormalities dichroism
ARIA-E	Vasogenic edema ARIA
ARIA-H	Hemorrhagic ARIA
BBB	Blood–brain barrier
CAA	Cerebral amyloid angiopathy
CC	Corpus callosum
DTI	Diffusion tensor imaging
Fc	Fragment crystallizable
FA	Fractional anisotropy
mAbs	Monoclonal antibodies
MRI	Magnetic resonance imaging
MTX	Matrix size
NTg	Non-transgenic mice
RARE	Multislice rapid acquisition with relaxation enhancement sequence
ROIs	Regions of interest
scFv	Single-chain variable fragment
T_2_w	T_2_-weighted
TE_eff_	Effective echo time
TR	Repetition time
VOI	Volume of interest
*λ*_||_	Axial diffusivity
*λ*_⊥_	Radial diffusivity
