# Supplementary material for: ScFv-h3D6 Prevents Bapineuzumab-Induced Hemorrhagic Events in the APP23 Mouse Model of Alzheimer’s Disease
Source: Biomolecules. 2025 Nov 15;15(11):1602. doi: 10.3390/biom15111602 (PMC12650597; doi:10.3390/biom15111602)
Supplement: Supplementary file 1 [file biomolecules-15-01602-s001.zip › biomolecules-3937663-supplementary.pdf]

Supplementary Information (SI)

Title: ScFv-h3D6 Prevents Bapineuzumab-Induced Hemorrhagic Events in the APP23 Mouse Model of Alzheimer's Disease

Silvia Lope-Piedrafita[1,2,3#*], Gabriel Serra-Mir[4#], Paula Melón[4], Anna Bonaterra-Pastra[5]; Mar Hernández-Guillamon[5], and Sandra Villegas[4*]

[1] Unitat de Biofísica, Departament de Bioquímica i Biologia Molecular, Facultat de Medicina, Universitat Autònoma de Barcelona, Bellaterra, 08193 Barcelona, Spain.

[2] Servei de Ressonància Magnètica Nuclear, Universitat Autònoma de Barcelona, 08193, Cerdanyola del Vallès, Spain.

[3] Centro de Investigación Biomédica en Red-Bioingeniería, Biomateriales y Nanomedicina (CIBER-BBN), Universitat Autònoma de Barcelona, 08193, Cerdanyola del Vallès, Spain.

[4] Protein Design and Immunotherapy Group, Departament de Bioquímica i Biologia Molecular, Facultat de Biociències, Universitat Autònoma de Barcelona, Bellaterra, 08193 Barcelona, Spain.

[5] Neurovascular Research Laboratory, Vall d'Hebron Research Institute, Universitat Autònoma de Barcelona, Barcelona, Spain.

* Corresponding author: Silvia.Lope@uab.cat ORCID: 0000-0002-8127-6425, and Sandra.Villegas@uab.cat ORCID: 0000-0001-8644-4304

# Both authors contributed equally to this work

Short Title: ScFv-h3D6 is safer than bapineuzumab

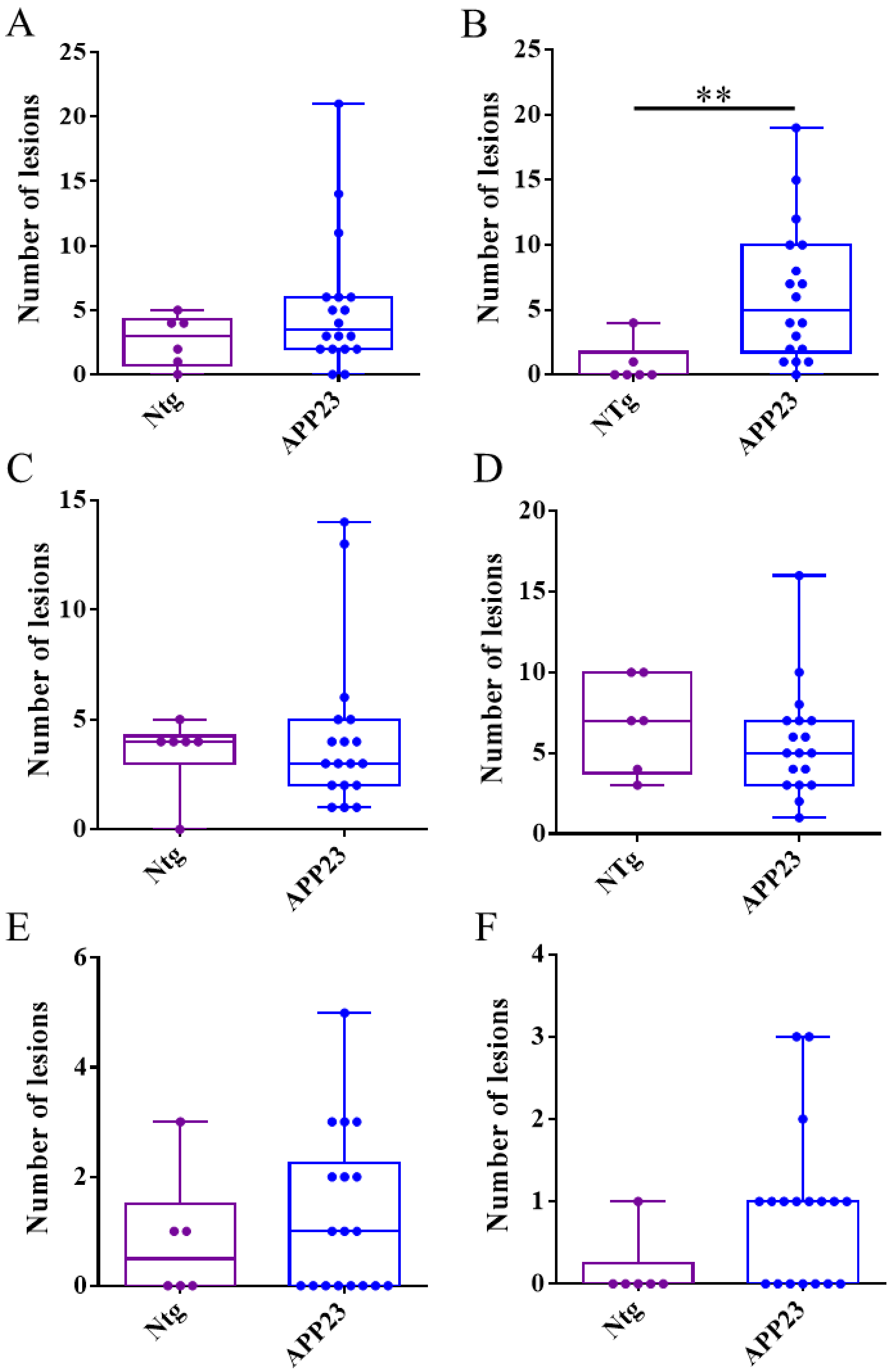

Figure S1. Quantification of the number of cerebral microhemorrhages from T_2*w images in different brain regions prior to treatments. (**A-E**) Number of cerebral lesions in NTg (purple) and APP23 (blue) mice prior to treatments in the right cortex (**A**), left cortex (**B**), right striatum/thalamus (**C**), left striatum/thalamus (**D**), right hippocampus (**E**), and left

hippocampus (**F**). Data are expressed as medians in box plots, and whiskers represent the minimum and maximum values. Statistical differences were assessed with the non-parametrical Mann–Whitney U test. * Significant differences $p \leq 0.05$; # Marginal significance $p \leq 0.1$; and § $r \geq 0.61$.

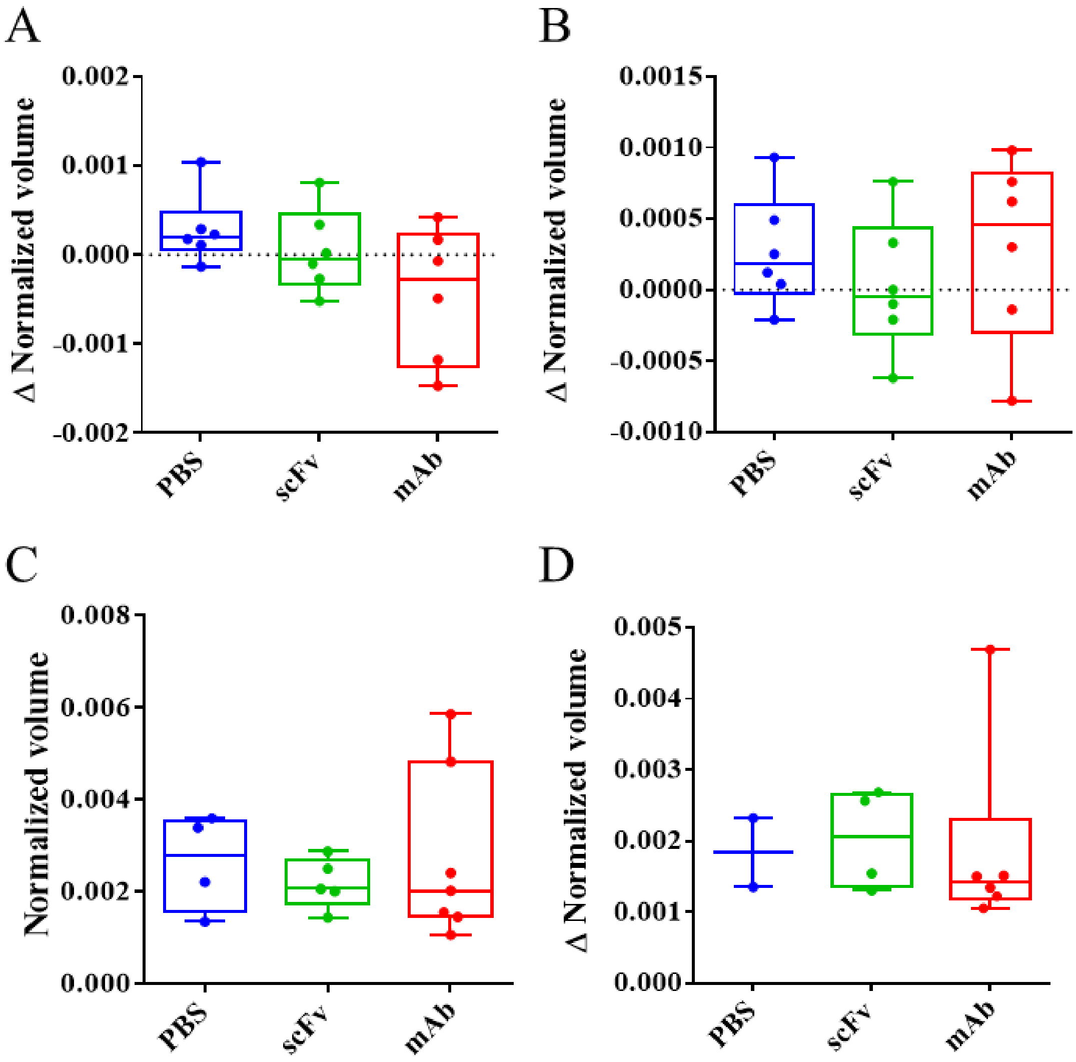

Figure S2. Determination of the volume of existing cerebral microhemorrhages from T_2*w images after treatments. (**A**) Volume increase after the first administration in lesions already present at the beginning of the experiment. (**B**) Volume increase after the second administration in lesions already present at the beginning of the experiment. (**C**) Volume of new lesions caused by the first administration. (**D**) Volume increase in new lesions caused by the second administration. Data are expressed as medians in box plots, and whiskers represent the minimum and maximum values. Statistical differences were assessed with the non-parametrical Mann–Whitney U-test. * Significant differences $p \leq 0.05$; # Marginal significance $p \leq 0.1$; and § $r \geq 0.61$.
